# Fatigue in patients with low grade glioma: systematic evaluation of assessment and prevalence

**DOI:** 10.1007/s11060-017-2454-4

**Published:** 2017-05-24

**Authors:** Ellen M. P. van Coevorden-van Loon, Marijke B. Coomans, Majanka H. Heijenbrok-Kal, Gerard M. Ribbers, Martin J. van den Bent

**Affiliations:** 1 Rotterdam Neurorehabilitation Research (RoNeRes), Rijndam Rehabilitation Center, PO Box 23181, 3001 KD Rotterdam, The Netherlands; 2 Department of Rehabilitation Medicine, Erasmus MC, Rotterdam, The Netherlands; 3 Department of Neurology/Neuro-oncology, Erasmus MC Cancer Institute, Rotterdam, The Netherlands

**Keywords:** Fatigue, Low-grade glioma, Assesssment, Prevalence

## Abstract

Fatigue is the most prevalent and disabling symptom in cancer patients. Yet, scientific literature on this topic is scarce and reports disparate results. This study systematically reviews how fatigue is assessed in patients with low-grade glioma and evaluates its prevalence in LGG patients. A systematic literature search was performed in PubMed, Embase and PsychINFO for articles reporting on fatigue in patients with LGG. Two reviewers independently extracted data from selected articles. Inclusion criteria were: (1) patients with suspected or confirmed LGG; (2) fatigue was assessed as primary or secondary outcome measure; (3) age≥ 18 years; (4) full-length article written in English or Dutch. In total, 19 articles were selected, including 971 patients. Seven self-assessment instruments were identified. Prevalence rates ranged from 39 to 77%. Fatigue was found to be a common side effect of treatment. The prevalence rates ranged from 20 to 76% when fatigue was reported as a mild or moderate side effect and fatigue was prevalent in 4% when reported as a severe side effect. Fatigue is a common problem in LGG patients that warrants more therapeutic and scientific attention. Gaining deeper insight in the underlying mechanisms of fatigue is essential in targeting therapy to individual patients.

## Introduction

Fatigue, characterized by feelings of tiredness, weakness and lack of energy, is the most frequently reported symptom of cancer. In all different types of cancer fatigue is common, up to 99% of the patients experience fatigue during the treatment and follow up [1–3]. Fatigue is the most important cause of loss of quality of life both for the patient and the care giver [4, 5]. Cancer-related fatigue is defined as a “persistent, subjective sense of tiredness related to cancer and cancer treatment that interferes with usual functioning” [6]. Despite its impact, fatigue in patients with cancer is underreported, underdiagnosed and undertreated [6]. Fatigue is a multidimensional concept. The National Comprehension Cancer Network (NCCN) has made an overview of coherent factors of the multidimensional concept of cancer-related fatigue, including: tumor-related factors and complications, comorbid conditions, psychological symptoms associated with the underlying tumor treatment, side effects of other medication, iatrogenic factors and psychological/behavioural factors [5].

This study focuses at patients with low grade glioma (LGG), defined as a grade I/II primary brain tumor arising from glial cells of the central nervous system including astrocytoma, oligodendroglioma, ependymoma or mixed glioma (oligoastrocytoma) according to the World Health Organization (WHO) [7]. Patients suffering from LGG have a median life expectancy of 5–15 years thanks to surgery, radiation and chemotherapy [2, 8–10]. There are few studies on the prevalence of fatigue in LGG patients. Proportions of fatigued in glioma patients vary from 39 to 77% [4, 11] which may be explained by differences between assessment methods, patient populations, and definitions of fatigue.

With the increasing survival time there is a growing need for development and improvement of treatment programs for fatigue in LGG patients which should be based on the currently available evidence [12, 13]. Therefore, the current systematic review was set up (1) to evaluate how fatigue is assessed in LGG patients, (2) to assess the prevalence of fatigue.

## Methods

A systematic search was performed in PubMed, Embase and PsychInfo in March 2016 using the search strategy presented in Table 1. After study selection, reference lists of all selected studies were examined.

**Table 1:** Search strategy

Data source	Search terms
Pubmed	((Glioma[mh] OR glioma*[tiab] OR astrocytoma*[tiab] OR ependymoma*[tiab] OR oligodendroma*[tiab]) AND (low grade*[tiab] OR grade I*[tiab] OR grade II*[tiab] OR grade 1*[tiab] OR grade 2*[tiab] OR grade1*[tiab] OR grade2*[tiab])) AND ((fatigue*[tiab]))
Embase	‘glioma’/exp OR glioma OR ‘astrocytoma’/exp OR astrocytoma OR glioma*:ab,ti OR astrocytoma*:ab,ti OR ependymoma*:ab,ti OR oligodendroma*:ab,ti AND (‘low grade’:ab,ti OR ‘grade i’:ab,ti OR ‘grade ii’:ab,ti OR ‘grade 1’:ab,ti OR ‘grade 2’:ab,ti OR ‘grade1’:ab,ti OR ‘grade2’:ab,ti) AND fatigue*:ab,ti
PsychINFO	(OR) glioma* astrocytoma* ependymoma* oligodendroma* (AND) (low grade) (AND) fatigue*

### Study selection

**Fig. 1:**
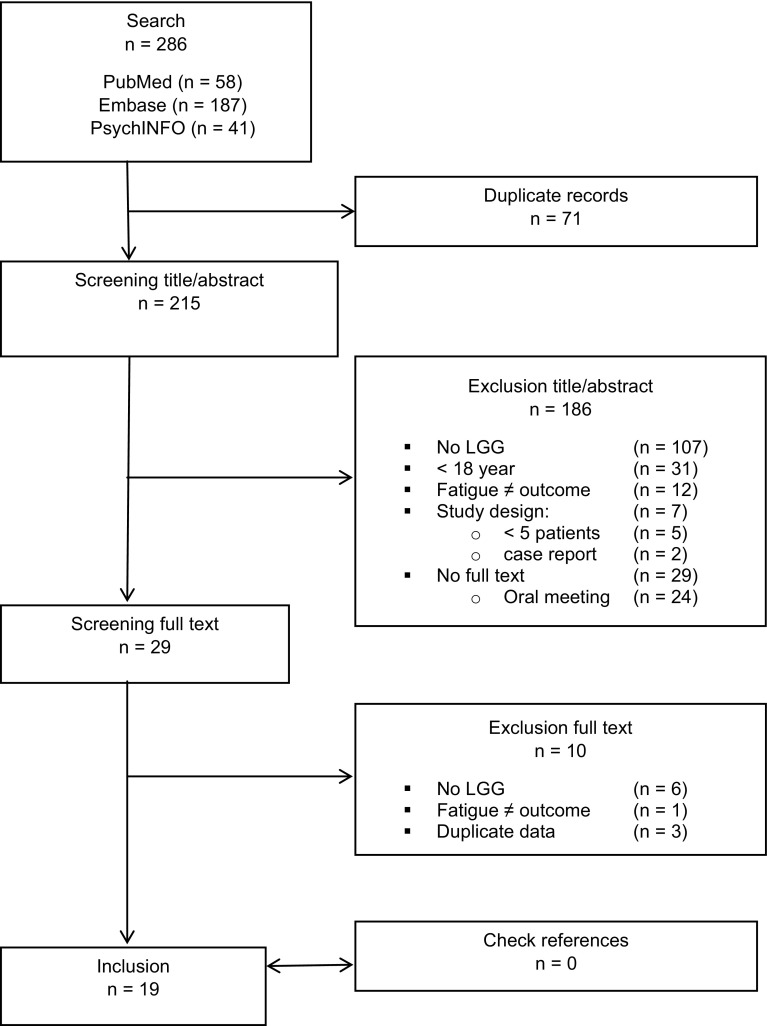
Flowchart of study selection

All titles and abstracts were screened independently by two investigators using the following criteria: (1) the study population included patients with suspected or confirmed LGG, (2) fatigue was reported as an outcome measure, (3) patients were ≥18 years of age at the time of diagnosis and (4) the publication was an original full-length manuscript in a peer-reviewed journal, written in English or Dutch. Studies including less than five patients and studies reporting duplicate data of previous studies were excluded. After a first selection of articles based on title/abstract, the full text manuscripts were retrieved and carefully assessed and evaluated according to the in- and exclusion criteria (Fig. 1). In case the authors disagreed, a third reviewer was consulted.

### Data extraction

After the final selection, two authors independently extracted data from the selected articles using a standard data extraction form. In case of disagreement, a third reviewer was consulted. Studies were differentiated in studies with fatigue as primary or secondary outcome (group 1) and studies in which fatigue was only reported as a side effect of a (new) treatment therapy (group 2). In cases of missing data the corresponding authors were contacted by email [14–23]. The results of responding authors were included [14, 15, 21].

### Data synthesis

The focus of this systematic review was to study instruments used for the assessment of fatigue of patients with LGG in a qualitative way and to gain to more insight in the prevalence of fatigue. Therefore, the results of this review are presented in tables using descriptive statistics.

## Results

The initial search identified 286 articles. After removal of duplicates in the three databases, the titles/abstracts of 215 studies were evaluated according to the inclusion criteria. A large number of articles (n = 107) were excluded because no LGG patients were included. Also, a relatively large number of articles were found to be abstracts of (oral) conference meetings (n = 24). Ultimately, 29 articles were screened in full text. The final dataset consisted of 19 articles that met all criteria. The characteristics of these studies are outlined in Table 2. A flowchart of the study selection is shown in Fig. 1.

**Table 2:** Main study characteristics of the included studies

Article references	A (n)	O (n)	OA (n)	E (n)	Oth (n)	U (n)	N	Sex men (%)	Age mean (±SD)	Tx (%)	TPD years (Mean±(SD)) (Median(range))^e^	Fatigue (outcome) 1, 2nd, S
Lee et al. [18]	NR	NR	NR	NR	NR	NR	5	NR	NR	NR	NR	1st
Struik et al. [11]	39	10	5	0	0	0	54	52	48 (±11.8)	S (69) RT (54) CT (NR)	15 (±4.0)	1st
Cheng et al. [16]	17	7	9	2	3	0	38	NR	NR	Pre Tx	NR	2nd
Dutzmann et al. [17]	0	0	0	51	0	0	51	NR	NR	S (51) RT (22)	NR	2nd
Gehring et al. [15]	70	45	20	0	12	0	147^a^	56	43.9 (±9.8)	S (59) RT (56) CT (7)	7.4 (±5.9)^b^	2nd
Gustafsson et al. [4]	21	7	4	4	1	0	37	69	47 (±14)	S (74) RT (59) CT (21)	10 (<1–47)^c^	2nd
Jakola et al. [29]	29	16	10	0	0	0	55	55	41 (±13)	S (82) RT (46) CT (33)	NR	2nd
Jones et al. [22]	NR	NR	NR	NR	NR	NR	10	60	40 (±8)	S (90) RT (NR) CT (NR)	NR	2nd
Kiebert et al. [30]	NR	NR	NR	0	0	0	345	57	NR	S (NR) RT (100)	NR	2nd
Shaw et al. [19]	12	NR	NR	NR	NR	NR	NR	54	45	NR	NR	2nd, S
Taphoorn et al. [34]	26	15	0	0	0	0	41	59	39.9 (±13)	S (61) RT (49)	3.5 (±2.7)	2nd
Chamberlain et al. [36]	0	0	0	25	0	0	25	60	48 (±10.9)	S (100) RT (100) CT (100)	NR	S
Duerinck et al. [20]	3	1	1	0	0	0	5	NR	NR	NR	NR	S
Hauswald et al. [50]	10	0	1	0	0	0	11	45	39 (±7.6)	S (36) RT (100) CT (18)	NR	S
Maquilan et al. [51]	9	2	4	0	2	0	17	39	NR	S (87) RT (100) CT (NR)	NR	S
Møller et al. [21]	8	2	2	0	0	0	12	67	49.5 (±10.6)	S (42) RT (100) CT (100)	NR	S
Okada et al. [38]	11	3	9	0	0	0	23	52	39 (±8.7)	S (NR) RT (9) CT (13)	NR	S
Pouratian et al. [37]	1	15	6	0	3	0	25	48	48 (21–76)^c^	S (56) CT (100)	NR	S
Taal et al. [23]	NR	NR	NR	NR	NR	NR	16	?	NR	NR	NR	S

*A* astrocytoma, *O* oligodendroma, *OA* oligoastrocytoma, *E* ependyoma, *Ot* other type of LGG, *U* histological unconfirmed tumor patients, *Tx* type of treatment, *CH* chemotherapy, *S* partial or complete resection, *RT* radiotherapy, *TPD* time post diagnosis, *Pre-Tx* pre-treatment, *S* fatigue measured as side effect, *NR* not reported in article

^a^Analysis on fatigue was reported in 146 patients

^b^Time since first reported symptoms

^c^range

### Study population

Nineteen studies encompassed a total number of 917 LGG patients. Differentiation in type of glioma was reported for 886 patients (97%). The most common diagnosis was astrocytoma (55%), followed by oligodendroglioma (22%), oligoastrocytoma (12%), ependymoma (9%) and other/unknown type of LGG (2%). The mean age of the total population was 41.9 years. A description of the reported time post-diagnosis was available in 36% of the patients and ranged from 3.5 to 15 years. In 90% of the patients a complete description of treatment was provided. One study reported results prior to treatment [16]. In all other included studies, patients underwent some type of treatment. Most of the patients underwent partial or complete surgery (80%), followed by radiotherapy (68%), chemotherapy (11%) or a combination. An overview of the study population characteristics reported in the included article is shown in Table 2.

### Results

In 12 out of 19 studies fatigue was a primary or secondary outcome measure, defined as ‘Group 1’, representing 85% (n = 753) of all patients. In eight studies fatigue was reported in the outcome category measure of adverse events. In that category, it was reported as a side effect of a treatment under study. These studies are defined as ‘Group 2’, representing 15% (n = 134) of all patients. One study measured fatigue both as a primary outcome and as a side effect of treatment [19].

### Measurements

In group 1 seven self-assessment instruments could be identified. Two studies used two or more instruments [18, 19]. Table 3 shows a short description and the internal consistency of the measurement instruments.

**Table 3:** Measurement instruments, characteristics and properties

Measurement instrument	Primary outcome	Short description	Items (nF)/nT	No of fatigue subscales	Internal con	References
Cancer Fatigue Scale (CFS)	Fatigue	Measuring three dimensions of fatigue Physical fatigue, cognitive fatigue, activity-related fatigue	15/15	3	0.88	[18]
Checklist individual strength (CIS)	Fatigue	Measuring four dimensions of fatigue; subjective fatigue, concentration, motivation, activity, total score	20/20	4	0.90	[11]
Multidimensional fatigue inventory (MFI)	Fatigue	Measuring five dimensions of fatigue general fatigue, physical fatigue, mental fatigue, reduced motivation, and reduced activity	20/20	5	0.84	[14]
Brief fatigue inventory (BFI)	Fatigue	Measuring the severity of fatigue and fatigue-related impairment in cancer patients	9/9	1	0.96	[18]
European Organization for Research and Treatment of Cancer Quality of Life Questionnaire C30 (EORTC QLQ-C30)	HRQoL	Measuring health related QoL. The questionnaire includes a three items fatigue subscale	3/30	1	0.80–0.85	[4, 16, 17, 29, 30]
Functional Assessment of Cancer Therapy–Fatigue Scale(FACT–F)	QoL	Measuring QoL in five domains; Physical well-being, emotional wellbeing, functional well-being, social/family, fatigue	13/40	1	0.95	[18, 22]
Profile of Mood States (POMS)	Mood	Measuring fatigue as subdomain total of scale with six domains of mood; Depression, anxiety, anger, subjective confusion, fatigue, and vigor	7/32	1	0.90–0.94	[19, 34]
The National Cancer Institute Common Terminology Criteria for Adverse Events (NCI CTC AE) version 2.0/3.0/4.0	Adverse effects	Grading system for the adverse effects (AE’s) of cancer treatment: grade 1 mild AE, grade 2 moderate AE, grade 3 severe AE, grade 4 life-threatening or disabling AE	NA	NA	NA	[19–21, 23, 36–38, 50, 51]

*nT* total numbers of items of the measurement, *nF* total numbers of items in the measurement about fatigue, *Con* consistency, *HRQoL* Health related quality of life, *QoL* Quality of life, *NA* not applicable

Three multidimensional fatigue scales were used in the articles: (1) the Cancer Fatigue Scale (CFS), (2) the checklist individual strength (CIS), and (3) the multidimensional fatigue inventory (MFI-20). The CFS consists of 15 items measuring physical, cognitive and activity-related fatigue [24]. One included study used the CFS [18]. The CIS consists of 20 items covering fatigue, fatigue severity (8 items), concentration problems (13 items), reduced motivation (4 items), and reduced activity (3 items) [25]. The CIS was used in one study [11]. The MFI-20 is a 20-item scale designed to evaluate five dimensions of fatigue: general fatigue, physical fatigue, reduced motivation, reduced activity, and mental fatigue [26]. The MFI was used in one included study [14]. The CFS, CIS and MFI-20 are used across different patient populations, including cancer patients [1, 24, 26]. One unidimensional fatigue scale was used in the included articles: the brief fatigue inventory (BFI). The BFI is a nine-item instrument to assess fatigue on a rating scale. The BFI was developed for the rapid assessment of fatigue severity in cancer patients [27]. The scale was used in one article [18].

In three multidimensional instruments, fatigue was measured as a subdomain of health-related quality of life or mood: (1) The European Organization for Research and Treatment of Cancer Quality of Life Questionnaire (EORTC QLQ-C30), (2) The Functional Assessment of Cancer Therapy–Fatigue Scale (FACT–F) and (3) The Profile of Mood States (POMS). The European Organization for Research and Treatment of Cancer Quality of Life Questionnaire (EORTC QLQ-C30) is a quality-of-life scale that includes a three-item fatigue subscale which has been widely used as an independently validated fatigue measure across different oncological populations. It was specifically designed for use in oncology [28]. Five included studies used the EORT QLQ C30 [4, 16, 17, 29, 30]. The Functional Assessment of Cancer Therapy–Fatigue (FACT–F) subscale is part of a collection of quality of life questionnaires targeting the management of chronic illness. It consists of 13 items and is frequently used in cancer populations [31, 32]. The FACT–F was assessed in two included studies [18, 22]. The Profile of Mood States (POMS) contains several scales including a fatigue subscale of seven items which have been used both in cancer and non-cancer populations [33]. Two included studies used the POMS [19, 34].

In all studies reporting fatigue as side effect of treatment, the National Cancer Institute Common Terminology Criteria for Adverse Events (NCI CTCAE) was used. The NCI CTCAE is a set of standardised definitions for adverse events and describes the severity of organ toxicity for patients receiving cancer therapy [35]. The NCI CTCAE describes fatigue as a disorder characterized by a state of generalized weakness with a pronounced inability to summon sufficient energy to accomplish daily activities. In this review, the identified articles used version 2.0, 3.0 or 4.0. The adverse events (AE’s) are defined based on grades one (mild), two (moderate), three (severe), four (life-threatening) and five (death related to AE).

### Prevalence

In group 1, the prevalence of fatigue was provided in three studies representing 19% of the total of included patients (146/783) [4, 11, 29]. The reported prevalence of fatigue ranged from 39% (long term surviving LGG patients experiencing severe fatigue) to 77% (feeling tired during the last week from “a little” to “very much”). Other results related to fatigue as an outcome measure were reported in 87% of the included patients (679/783). These results are shown in Table 4.

**Table 4:** Prevalence and severity of fatigue

Article	Primary outcome study	Reported results on fatigue LGG
Lee et al. [18]	Fatigue	NR
Struik et al. [11]	Fatigue	39% severe fatigue^a^
Cheng et al. [16]	HRQoL	NR
Dutzmann et al. [17]	HRQoL	NR
Gehring et al. [14]	Cognitive functioning	Above average score on scale “mental fatigue”
Gustafsson et al. [4]	Function, HRQoL, coping	77% feeling tired during the last week from a little to “very much”^a^
Jakola et al. [29]	Overall survival, HRQoL	44% experienced symptoms in at least one of the fatigue related questions^a^
Jones et al. [22]	Functional performance measures post-surgery	NR
Kiebert et al. [30]	HRQoL	Low median scores were found for fatigue
Shaw et al. [19]	Cognitive functioning, mood, and QoL	NR
Taphoorn et al. [34]	QoL and cognitive functioning	LGG patients scored higher on the subscales fatigue than did control subjects
Chamberlain et al. [36]	Side effect of CT	FG 1/2:20%
Duerinck et al. [20]	Side effect of CT	NR
Møller et al. [21]	Side effect of CT	NR
Pouratian et al. [37]	Side effect of CT	FG 1/2:76%
Taal et al. [23]	Side effect of CT	NR
Okada et al. [38]	Side effect of IT	FG 1:35%, FG 2:52%, FG 3:4%
Hauswald et al. [50]	Side effect of RT	NR
Maquilan et al. [51]	Side effect of RT	NR

*NR* not reported, *FG* fatigue grade of toxicity, *CT* chemotherapy, *IT* immunotherapy, *RT* radiotherapy

^a^Reported prevalence on fatigue

In group 2, a detailed description of fatigue as a side effect of treatment was provided in three studies, representing 54% (73/134) of the patients [36–38]. The reported prevalence as a side effect of treatment ranged from 20 to 76% when fatigue was reported as a mild (grade 1) or moderate (grade 2), side effect. When reported as a severe (grade 3) side effect, fatigue was prevalent in 4% of the patients.

## Discussion

This systematic review shows a variety of instruments that are used to measure fatigue in LGG patients. We identified seven self-assessment instruments (CFS, CIS, MFI, BFI, EORTC QOL-C30, FACT, POMS). All scales were used in a number of different cancer populations and have a good internal validity. Fatigue was the primary outcome only in two studies. In all other studies fatigue was a secondary outcome. When fatigue was reported as a side effect of treatment, the National Cancer Institute Common Terminology Criteria for Adverse Events is uniformly used. This is in agreement with the recommendation of the National Cancer Institute [35].

The EORTC QOL-C30 is the most frequently used instrument included in this review. Unidimensional instruments or subscales measure the severity of perceived fatigue and tend to be short and easy-to-use. Also, they have the most robust psychometric data to support their use since they are widely used: the FACT and the EORTC QLQ-C30 have been used in over 10.000 patients [39, 40]. However, fatigue is a multidimensional concept involving mood disorders, anxiety, cognitive disorders and physical distress [5]. When the goal is to gain insight in fatigue, rather than only assess the level of perceived fatigue, a multidimensional fatigue instrument provides a more comprehensive view [3]. This is line with the Dutch oncology guidelines, that recommend to use the multidimensional fatigue instrument (MFI) for measuring cancer-related fatigue [41]. The MFI is considered to be the most frequently used multidimensional fatigue instrument in clinical care in the Netherlands and was found to be reliable, valid, easy to handle and responsive [26, 39, 41, 42]. Additionally, it must be taken into account that even multidimensional fatigue instruments will only gain insight in the subjective experience of fatigue since fatigue is self-reported. The patient’s representations of their physical and cognitive functioning might not always correspond with objective measurement of physical activity, performance and cognitive functioning [43].

Fatigue is not only the most common symptom in cancer patients, but also a typical disabling symptom in neurological disorders such as stroke [44]. One theory of fatigue in patients with traumatic brain injury and stroke patients is the ‘cognitive coping hypothesis’. This hypothesis states that patients with brain injury have to put in more effort to accomplish tasks, compared to non-injured individuals [45, 46]. For patients, this means that they tire more easily and they need longer to recuperate from fatigue then before the injury [45]. We think this theory can be one of the explanations for fatigue in LGG patients. Gehring et al. [15] found result that lower ratings of cognitive function in LGG patients were associated with self-reported mental fatigue, measured with the MFI. Research to analyse the factors of fatigue in low grade patients with subjective and objective measurement outcomes will be useful in developing individualized rehabilitation treatment programs in LGG patients with fatigue.

Because fatigue was a primary outcome in only two studies, we found limited information on prevalence rates. However, the included studies do show that fatigue seems to be a frequent problem prior to treatment [16], during radio- and chemotherapy [17, 30] and in the long term after treatment [11]. This is in line with patients with brain tumours of different origins than LGG [1, 47]. Unfortunately, this review was not able to distinguish the different contributions in terms of fatigue from different treatment modalities. It can be stated that fatigue is a common side effect of these treatments, as is known from the literature [48]. Fatigue is both a frequent symptom of LGG as a side effects of its treatment and the relative effects of the disease versus treatment are difficult to distinguish. This is a major challenge in clinical decision making, weighing the benefits against the adverse effects of treatment. In newly diagnosed LGG patients the risks and benefits of treatment strategies including resection, radiation, chemotherapy or “watchful waiting” are recommended to be individually weighed [41, 49].

### Conclusion

Despite the growing awareness of cancer-related fatigue, there is still a lack of knowledge of the exact pathophysiology of fatigue and the underlying mechanisms of fatigue in LGG patients. This review shows that fatigue is a common problem in LGG patients, both as a disease symptom and as a side effect of treatment. Measurement of fatigue is complex and multiple instruments need to be used for proper assessment. Incorporation of the patients’ perspective (patient reported outcome) with a multidimensional fatigue instrument is in line with the current guidelines. Additionally, it must be taken into account that even multidimensional fatigue instruments not yield sufficient insight in potential causes or consequences of fatigue, like a decrease in physical and cognitive performance. We suggest research to analyse the factors of fatigue in low grade patients with subjective and objective measurement outcomes to developing individualized rehabilitation treatment programs in LGG patients.
